# Antimicrobial Resistance Patterns in Women with Positive Urine Culture: Does Menopausal Status Make a Significant Difference?

**DOI:** 10.1155/2017/4192908

**Published:** 2017-04-13

**Authors:** Pawel Miotla, Katarzyna Romanek-Piva, Michal Bogusiewicz, Ewa Markut-Miotla, Aneta Adamiak, Andrzej Wróbel, Małgorzata Zebrowska, Sara Wawrysiuk, Katarzyna Mendyk, Ewa Rechberger, Agata Jakubczak, Tomasz Rechberger

**Affiliations:** ^1^2nd Department of Gynaecology, Medical University of Lublin, Lublin, Poland; ^2^Department of Paediatric Pulmonology and Rheumatology, Medical University of Lublin, Lublin, Poland; ^3^Medical University of Lublin, Lublin, Poland; ^4^CM Luxmed, Lublin, Poland

## Abstract

*Aim*. Urinary tract infection (UTI) is considered one of the most common bacterial infections in women. The aim of this study was to investigate the types of uropathogens present, as well as the degree of antimicrobial drug resistance seen among premenopausal (*n* = 2748) and postmenopausal (*n* = 1705) women with uncomplicated UTI.* Methods*. Urinary samples (*n* = 4453) collected from women with UTI were analyzed in terms of uropathogens present. These were considered as positive if bacterial growth was ≥10^5^ colony forming units (CFUs)/mL. Susceptibility and resistance testing for commonly used antibiotics was subsequently assessed.* Results*. The most common uropathogens cultured from urine samples were* Escherichia coli* (65.5%), followed by* Enterococcus faecalis* (12.2%),* Klebsiella pneumoniae* (4.7%), and* Proteus mirabilis* (4.2%). The resistance to ampicillin exceeded 40%, independently of menopausal status. Of note, resistance to ciprofloxacin exceeded 25% among postmenopausal patients. Moreover, resistance of all uropathogens to commonly used antimicrobials was significantly higher in postmenopausal women.* Conclusion*. Due to the high resistance rate, ampicillin, ciprofloxacin, and the trimethoprim/sulfamethoxazole combination should be avoided in treating postmenopausal women affected by UTI without being indicated by initial urine culture report. Finally, cephalexin and cefuroxime are promising alternatives as initial treatment in postmenopausal women.

## 1. Introduction

Urinary tract infection (UTI) is a very common bacterial disease in women of all ages. Indeed, it has been reported that nearly 1 out of 3 women has at least 1 episode of UTI requiring antimicrobial therapy by the age of 24 years [1]. Moreover, the lifetime risk for UTI in women is calculated as greater than 50% [2]. Robinson et al. [3] have calculated that the risk of UTI in women is 14 times higher than in men. The majority of UTI in women are strictly limited to the bladder. However, the variety of clinical manifestations ranges from simple, uncomplicated cystitis to severe urosepsis. The most common symptoms of UTI in premenopausal women are urinary urgency, frequency, and dysuria [4]. These symptoms are very similar to storage symptoms observed in overactive bladder patients, and, therefore, it might raise the need for differential diagnosis [5]. Postmenopausal women with UTI may report nocturia, malaise, urinary incontinence, or odorous urine [4, 6].

A self-reporting questionnaire for clinical diagnosis of uncomplicated cystitis (Acute Cystitis Symptom Score (ACSS)) can be a useful tool in the assessment of UTI in women [7]. As a majority of affected women initially attend the offices of general practitioners (GPs), GPs are crucial in the proper recognition and in the provision of adequate treatment of UTI, and rapid initiation of appropriate empirical treatment requires a good knowledge of epidemiological data concerning the sensitivity of uropathogens to antibacterial agents. Among common risk factors besides that of sexual activity, general health status, diabetes, pregnancy, and history of UTI in past are commonly cited. Community-associated UTI occurs mainly in the female population, and in self-reported surveys, almost 10.8% of women have declared to have had UTI symptoms within last 12 months [2]. In postmenopausal women, additional reasons for the increased risk of UTI are anatomical (cystocele; uterine prolapse), functional (voiding disturbances; urinary incontinence), and hormonal (vaginal atrophy due to hypoestrogenism) changes [4, 6].

The aim of our study was to evaluate total drug resistance in urine sample cultures collected in outpatient clinics from adult women with nonrecurrent, uncomplicated UTI. The intent is to simplify efficient decision-making in either general or gynaecological practice.

## 2. Materials and Methods

The protocol of this study was approved by our local institutional Ethical Committee. We evaluated 4453 positive urine samples collected from women who underwent urinalysis in ambulatory care between 2013 and 2015 due to symptoms of uncomplicated UTI. Urine samples were considered as positive when urine culture resulted in ≥ 10^5^ colony forming units (CFUs)/mL. In all collected samples, bacterial identification and susceptibility testing were performed at the same laboratory. Disk-diffusion tests were used for resistance and susceptibility testing with application of appropriate disks, including ampicillin (10 *μ*g), trimethoprim/sulfamethoxazole (11.25 *μ*g/23.75 *μ*g), ciprofloxacin (5 *μ*g), amoxicillin/clavulanic acid (20 *μ*g/10 *μ*g), nitrofurantoin (100 *μ*g), cephalexin (30 *μ*g), and cefuroxime (30 *μ*g). Extended-spectrum *β*-lactamase (ESBL) isolates were identified by specific polymerase chain reaction (PCR) and sequencing as described in the literature [8]. The obtained isolates were identified using standard methods, via a commercial bacterial identification system (Vitek 2 Compact, BioMérieux, France). Women with monomicrobial infection were grouped according to menopausal status (premenopausal: *n* = 2748 and postmenopausal: *n* = 1705). The differences in total antimicrobial resistance were calculated for all pathogens. Additionally, meticulous differences in resistance to ampicillin, trimethoprim/sulfamethoxazole, ciprofloxacin, amoxicillin/clavulanic acid, nitrofurantoin, cephalexin, and cefuroxime were calculated for the six most important pathogens. Statistical analysis was performed with Statistica 12.0 (Dell, Round Rock, Texas, USA), using the *χ*^2^ test. A *p* < 0.05 was considered statistically significant.

## 3. Results and Discussion

The most common pathogens found in the urine samples collected from the entire sample population were* Escherichia coli (E. coli) *[65.5%] followed by* Enterococcus faecalis (E. faecalis)* [12.2%],* Klebsiella pneumoniae (K. pneumoniae) *[4.7%], and* Proteus mirabilis (P. mirabilis)* [4.2%]. All details concerning isolates are summarized in Table 1. Contrary to previously published studies, we have observed a significant, and, therefore, clinically relevant shift of uropathogens types which were responsible for uncomplicated UTI cases. Previously, Ronald had reported that* E. coli *was responsible for approximately more than 80% of all cases of UTI [9]. This incidence is higher when compared to our results, 65.5%. It has been also reported that* Staphylococcus saprophyticus (S. saprophyticus)* was associated with 5–15% of all UTI, whereas in our study this uropathogen was cultured only in 1.0% of all samples [9, 10].

Data concerning drug resistance (independently of bacterial pattern) in urine culture samples in the analyzed groups of patients are presented in Table 2. Drug resistance was seen to increase with patients' age and menopausal status, and these correlations were statistically significant in all classes of antimicrobials (Table 2). What should be underlined is that the resistance to ampicillin exceeded 40%, independently of menopausal status. Of note, ciprofloxacin, one of the most frequently prescribed drugs for UTI, appeared to be ineffective in one-fourth of all postmenopausal patients.

The isolates of ESBL-producing* E. coli *were found to be significantly higher in postmenopausal women. Interestingly, a majority of ESBL-producing* E. coli *strains were resistant to ampicillin, cephalexin, and cefuroxime in the pre- as well as in the postmenopausal groups. What is more, the resistance rates of ESBL-producing* E. coli *to nitrofurantoin were 11.5% and 21.7% in the premenopausal and the postmenopausal groups, respectively (see details in Table 3). We have also observed high resistance rates of ESBL-producing* K. pneumoniae *to all analyzed classes of antimicrobials in the postmenopausal group. The statistical differences in resistance of the most common uropathogens to the investigated antimicrobials are summarized in detail in Table 3.

The empirical treatment of UTI depends on susceptibility data based on local antimicrobial resistance in a given community [10]. Still, current trends show increasing rates of resistance to various antimicrobial agents independently of pathogen all over the world. The effective initial treatment of UTI might prevent the rapidly increasing bacterial resistance to antimicrobial drugs [8, 10]. Hooton et al. [11] postulated in 2004 that increased use of fluoroquinolones would lead to decrease of efficacy to this class of antimicrobial drugs.

In a multicentre, international survey study (Antimicrobial Resistance Epidemiological Survey on Cystitis (ARESC)) the prevalence of pathogens and their susceptibility in women with cystitis was investigated. Herein, the authors found that* E. coli *was isolated from a majority of urine samples (76.7%) [12]. Due to their high prevalence in urine culture,* E. coli *strains had the major influence on drug resistance patterns in the entire population. Therefore, we have also focused on the resistance of* E. coli *to antimicrobial drugs. In our study,* E. coli *was also the predominant uropathogen, and it was cultured in 65.5% of all samples from the entire study group. Moreover,* E. coli *was significantly more frequently isolated in the postmenopausal group (69.6%), in comparison to the premenopausal group (63.0%). While it has been previously published that* E. coli *was responsible for 71.4% cases of UTI in the Polish population, the authors have observed a decrease of* E. coli *as a uropathogen associated with UTI, in comparison with their previous study (83.7%) which was published 15 years ago [13, 14].

We also found that ampicillin was a less effective drug for UTI treatment as more than 50% of all microbe cultures were resistant to this antibiotic. In addition, the resistance of* E. coli *strains to ampicillin was significantly higher in the postmenopausal group (53.8%). However, even in premenopausal women, this drug seemed to be ineffective. Indeed, 47% of all* E. coli *strains were resistant to ampicillin. In a study published by Chlabicz et al. [15],* E. coli* also showed a high rate (40%) of resistance to ampicillin.

Naber et al. [16] recommended treatment with cotrimoxazole (160/800 mg twice daily for 5 consecutive days) in communities in which the resistance rates of* E. coli* to this antimicrobial agent are lower than 20%. In this study, we observed that resistance of all pathogens to cotrimoxazole exceeded 27% in postmenopausal women. Of note, the resistance rates of* E. coli *to cotrimoxazole were 19.6% and almost 26% in both the premenopausal and postmenopausal groups, respectively. Based on a recommendation published by Naber et al., we should, hence, be cautious with empirical cotrimoxazole administration in women with uncomplicated UTI, because such therapy has been found to be ineffective in one-fourth of all postmenopausal women.

Schito et al. reported that the resistance rate of* E. coli* strains isolated from urine samples collected in Poland was 6.6% for ciprofloxacin [12]. In our study, the total microbe resistance for ciprofloxacin was significantly higher in the postmenopausal women's group (24.2%), compared to the premenopausal group (10.8%). Indeed, the resistance rate of* E. coli *strains for ciprofloxacin was 10.7% and 22.7%, respectively, in the premenopausal and the postmenopausal groups. A direct comparison between the ARESC study and our results (independently, with both the pre- or postmenopausal groups) shows an increase of* E. coli* resistance to ciprofloxacin. It is important to note that in the ARESC study,* E. coli *revealed susceptibility to ciprofloxacin in 91.7% of all participating countries [12]. Seven years later, Stefaniuk et al. reported that over 24% of all* E. coli *colonies have become resistant to ciprofloxacin in uncomplicated UTI and that the resistance rate has exceeded 41% in complicated UTI [13]. We have found in our study that resistance of all uropathogens to ciprofloxacin was greater than 24% in the postmenopausal group; however, in the premenopausal patients, this antimicrobial agent was ineffective only in 11% of all cases. Thus, almost 10.7% and 23% of isolated* E. coli *strains were resistant to ciprofloxacin in the premenopausal and postmenopausal groups, respectively. These findings are similar to the results published by Stefaniuk et al. in 2016 [13].

Our results suggest that ciprofloxacin should be administered only with caution as an empirical treatment in postmenopausal women, even in uncomplicated UTI.

With regard to this notion, a large, national study was conducted in the United States to analyze the outpatient prescribing practices for uncomplicated UTI in women in 2002–2011. Herein, the authors found that fluoroquinolones were the most frequently prescribed antimicrobials (49%) for uncomplicated UTI. Therefore, they have concluded that the overuse of fluoroquinolones should be decreased and that such effort would reduce the risk of antimicrobial resistance to this class of drugs in the future [17]. Interestingly, the use of ciprofloxacin can also lead to collateral damage to commensal microbiota, the role of which in the past was probably underestimated. Indeed, in patients with uncomplicated UTI and treated via ciprofloxacin administration, a significant reduction in the proportions of* Bifidobacterium*,* Alistipes*, and phylum* Firmicutes* in the gut flora was observed, and the recovery to normal gut microbiome took up to one month after treatment [18]. Furthermore, emerging discoveries have revealed that commensal urinary microbiota differs between a healthy population and that with various urologic disorders. It has, hence, been proposed that future treatment strategies should include the usage of intravesical probiotics to decrease both the rates of UTI and the resistance to antimicrobials [19].

In our study, we calculated that the rates of total resistance to amoxicillin/clavulanic acid (AMC) were 12.3% and 18.9% in the pre- and postmenopausal groups, respectively. Hence, more than 13% of* E. coli *isolates were resistant to this antimicrobial agent in postmenopausal women. In a previously published study, the resistance rates to AMC varied from 1.2% to 9.6% [11]. From this point of view, the results seen in our group can be considered as alarmingly high.

What is more, in our study, the resistance rates to nitrofurantoin of all uropathogens and* E. coli *in premenopausal women were 8.9% and 7.8%, respectively. In the postmenopausal group, the number of resistant* E. coli *strains was doubled (15.7%). Our results are consistent with Naber et al., who concluded that the resistance rate of* E. coli *to nitrofurantoin was below 10% in premenopausal women. Therefore, we concur with their recommendation of a 5-day therapy with nitrofurantoin for the treatment of uncomplicated UTI in premenopausal women [16].

In our study, we saw that the resistance rates of* E. coli* to cephalexin (LEX) were relatively low (5% and 8.9%) in both the pre- and postmenopausal groups, respectively. For all uropathogens, these rates were higher (8.4% and 13.5%), and this effect was associated with increased rates of resistance to LEX observed in ESBL-producing* E. coli *and ESBL-producing* K. pneumoniae *strains. Schito et al. published that the resistance rate of* E. coli* strains isolated from urine samples collected in Poland was 2.2% for cefuroxime [12].

In our study, the resistance of all bacterial pathogens to cefuroxime was significantly higher in the postmenopausal women's group (10.8%), when compared to the premenopausal group (5.7%). Indeed, we found that the resistance rates of* E. coli *strains for cefuroxime were 2.8% in the premenopausal group and 4.7% in the postmenopausal group. Based on a comparison of these data with that previously published in the ARESC study, we see an increase of* E. coli* resistance to cefuroxime [12]. The results of our study suggest that ampicillin should be definitely avoided in the initial treatment of UTI in women whose infection had not been indicated by urine culture results. In addition, even currently, commonly used therapy with ciprofloxacin or cotrimoxazole could be completely ineffective in one-fourth of all postmenopausal women. Therefore, these antimicrobial agents should be avoided in any empirical UTI treatment in these women. Furthermore, as genital urinary atrophy due to oestrogen depletion eases the progression of UTI, local oestrogen therapy for vaginal atrophy is recommended as a part of UTI treatment in postmenopausal patients [20].

## 4. Conclusions

A significant shift of uropathogens types responsible for uncomplicated UTI was observed in our study. Moreover, compared to recently published studies, we had observed an increase in resistance among bacterial uropathogens to commonly prescribed antimicrobial agents. Additionally, the resistance of pathogens to all investigated antimicrobials was significantly higher in the postmenopausal group. Furthermore, ampicillin, ciprofloxacin, and the trimethoprim/sulfamethoxazole combination should be avoided in treating postmenopausal women affected by UTI without being indicated by initial urine culture report. Among drugs of choice, cephalosporins such as cephalexin or cefuroxime can be recommended as initial treatment in postmenopausal women. Thus, the decision of initial therapy should be made based on the epidemiological updated recommendations, which should consider drug resistance patterns and the distribution of uropathogens in the local community.

## Figures and Tables

**Table 1:** Prevalence of most common pathogens isolated from urine samples in women with symptoms of urinary tract infection.

Pathogen isolated from urine sample	Entire population (*n* = 4453) *n* (%)	Premenopausal women (*n* = 2748) (mean age 32.2 ± 5.1 years) *n* (%)	Postmenopausal women (*n* = 1705) (mean age 61.3 ± 7.8 years) *n* (%)	Difference in isolates premenopausal versus postmenopausal (*χ*^2^)
*Escherichia coli*	2917 (65.5)	1730 (63.0)	1187 (69.6)	*χ* ^2^ = 20.7
				*p* < 0.001

*Enterococcus faecalis*	545 (12.2)	393 (14.3)	152 (8.9)	*χ* ^2^ = 28.4
				*p* < 0.001

*Klebsiella pneumoniae*	210 (4.7)	128 (4.7)	82 (4.8)	NS

*Proteus mirabilis*	185 (4.2)	107 (3.9)	78 (4.6)	NS

ESBL-producing *Escherichia coli *	75 (1.7)	26 (1.0)	49 (2.9)	*χ* ^2^ = 23.6
				*p* < 0.001

*Streptococcus agalactiae*	70 (1.6)	64 (2.3)	6 (0.4)	*χ* ^2^ = 27.0
				*p* < 0.001

ESBL-producing *Klebsiella pneumoniae *	59 (1.3)	39 (1.4)	20 (1.2)	NS

*Staphylococcus aureus*	49 (1.1)	38 (1.4)	11 (0.7)	*χ* ^2^ = 5.3
				*p* < 0.05

*Staphylococcus saprophyticus*	45 (1.0)	37 (1.4)	8 (0.5)	*χ* ^2^ = 8.1
				*p* = 0.005

Others (incidence ≤ 1% in entire population)	298 (6.7)	186 (6.7)	112 (6.5)	NS

ESBL: extended-spectrum *β*-lactamase.

**Table 2:** Total antimicrobial resistance of all uropathogens isolated from urine samples.

Group of antimicrobial agents	Resistance in entire population (%)	Resistance in samples collected from premenopausal women (%)	Resistance in samples collected from postmenopausal women (%)	Difference in resistance: premenopausal women versus postmenopausal women (*χ*^2^)
Ampicillin	45.9	42.4	51.4	*χ* ^2^ = 31.4
				*p* < 0.001
Trimethoprim/ sulfamethoxazole	22.3	18.9	27.2	*χ* ^2^ = 35.1
				*p* < 0.001
Ciprofloxacin	16.2	10.8	24.2	*χ* ^2^ = 117.7
				*p* < 0.001
Amoxicillin/ clavulanic acid	15.3	12.3	18.9	*χ* ^2^ = 25.1
				*p* < 0.001
Nitrofurantoin	12.5	8.9	18.0	*χ* ^2^ = 65.5
				*p* < 0.001
Cephalexin	10.5	8.4	13.5	*χ* ^2^ = 30.8
				*p* < 0.001
Cefuroxime	7.8	5.7	10.8	*χ* ^2^ = 31.1
				*p* < 0.001

**Table 3:** The resistance of selected uropathogens to antimicrobial agents.

Pathogen	Resistance to each antimicrobial agent (premenopausal (PRE) and postmenopausal (POST) groups) (%)														Difference in resistance to each antimicrobial agent: premenopausal versus postmenopausal group *p* value (*χ*^2^)						
	AMP		SXT		CIP		AMC		NIT		LEX		CXM		AMP	SXT	CIP	AMC	NIT	LEX	CXM
	PRE	POST	PRE	POST	PRE	POST	PRE	POST	PRE	POST	PRE	POST	PRE	POST							
*Escherichia coli*	47.2	53.8	19.6	25.9	10.7	22.7	10.3	13.4	7.8	15.7	5	8.9	2.8	4.7	<0.001 (12.2)	<0.001 (16)	<0.001 (75.6)	<0.001 (23.6)	<0.001 (44.4)	<0.001 (17.3)	<0.01 (7.2)
*Enterococcus faecalis*	0	0	N/A	N/A	12.5	33.3	7.7	N/A	0.5	4	0	N/A	0	N/A	NS	N/A	NS	N/A	<0.01 (8.9)	N/A	N/A
*Klebsiella pneumoniae*	97.6	96.1	7.9	20.7	7.1	18.8	9.3	20.0	50	88.9	4.8	18.8	3.9	12.2	NS	<0.01 (7.2)	=0.01 (6.5)	<0.05 (4.5)	NS	=0.001 (10.3)	<0.05 (4.5)
*Proteus mirabilis*	32.7	32.5	18.9	33.3	15.2	14.7	10.3	10.4	92.9	83.8	13.1	17.1	2.8	3.9	NS	<0.05 (4.5)	NS	NS	NS	NS	NS
ESBL-producing *Escherichia coli *	86.4	92.6	38.5	64.6	42.3	71.4	23.1	70.2	11.5	21.7	87.5	90.0	87.5	93.6	NS	<0.05 (4.7)	<0.05 (6.1)	=0.001 (15.3)	NS	NS	NS
ESBL-producing *Klebsiella pneumoniae *	100	100	46.0	52.9	28.2	65.0	50	61.5	71.4	75	34.5	45.5	41.7	65	NA	NS	=0.01 (7.4)	NS	NS	NS	NS

AMP: ampicillin, SXT: trimethoprim/sulfamethoxazole, CIP: ciprofloxacin, AMC: amoxicillin/clavulanic acid, NIT: nitrofurantoin, LEX: cephalexin, and CXM: cefuroxime.

ESBL: extended-spectrum *β*-lactamase.
